# Exposure of Young Children to Permethrin and Cypermethrin Insecticides in the Residential Environment

**DOI:** 10.3390/toxics12070477

**Published:** 2024-06-30

**Authors:** Siriporn Sirikanyaporn, Noppanun Nankongnab, Pornpimol Kongtip, Sukhontha Siri, William Alfred Suk, Susan Renee Woskie

**Affiliations:** 1 Department of Occupational Health and Safety, Faculty of Public Health, Mahidol University, 420/1 Rajvidhi Road, Bangkok 10400, Thailand; siriporn.skp@gmail.com (S.S.); pornpimol.kon@mahidol.ac.th (P.K.); 2 Public Health, Mahidol University, Amnatcharoen Campus, Amnatcharoen 37000, Thailand; 3 Department of Epidemiology, Faculty of Public Health, Mahidol University, Bangkok 10400, Thailand; sukhontha.sir@mahidol.ac.th; 4 Department of Environmental Sciences and Engineering, Gillings School of Global Public Health, The University of North Carolina at Chapel Hill, 135 Dauer Drive, Chapel Hill, NC 27599, USA; bill.suk09@gmail.com; 5 Department of Public Health, University of Massachusetts Lowell, 61 Wilder St., Lowell, MA 01854, USA; susan_woskie@uml.edu

**Keywords:** permethrin, cypermethrin, urinary pyrethroid metabolites, young children

## Abstract

The aims of this study were to evaluate the exposure to permethrin and cypermethrin of young children aged between 2 and 5 years in Nakhon Pathom and Sing Buri provinces, Thailand. A questionnaire that included general demographic information, household characteristics, insecticide usage and exposure-related behavior in children was used to interview parents or family caregivers. Permethrin and cypermethrin concentrations on floor surfaces and children’s hands, as well as their urinary metabolites, were analyzed by gas chromatography coupled with mass spectrometry. The results showed that permethrin and cypermethrin were detected in 62% and 83% of the children’s hand wipe samples, with geometric mean (GM) levels of 0.02 µg and 0.04 µg, respectively. Permethrin and cypermethrin were detected in 79% and 93% of floor surface wipe samples, with GM levels of 0.90 µg/m^2^, and 1.49 µg/m^2^, respectively. For children’s urine, the GM concentrations of cis- and trans-DCCA, 3-PBA, and total pyrethroid metabolites were 0.84, 0.31 and 1.23 nmol/g creatinine, respectively. This study found that household insecticide product usage and having a tile floor were associated with increased permethrin concentrations on the children’s hands and floor surfaces. However, cypermethrin concentrations on floor surfaces were significantly higher in families using aerosol insecticide sprays and insecticide products in the living room and bedroom. The predictors of the total pyrethroid, DCCA and 3-PBA metabolites are permethrin on children’s hands or floor surfaces and cypermethrin on floor surfaces.

## 1. Introduction

Concern over pesticide exposures in residential environments is increasing, especially with regard to children’s health. Previous studies have confirmed that children could be exposed to pesticides at home through various environmental pathways, such as consuming contaminated foods and water, inhaling air pollution, or dermal contact with house dust [1,2]. Due to their physiologic characteristics, such as a higher breathing and food consumption rate and larger skin surface area per body weight than adults [3], children may be at greater health risk than adults when exposed to environmental chemicals; these characteristics influence the internal dose of environmental contaminants in children. In addition, the behavior of young children, who crawl and spend time on the floor and frequently put their hands and objects into their mouths, may increase their pesticide exposure in the residential environment [3,4].

Pyrethroids are synthetic insecticides widely used for both agricultural and household insect control due to their lower mammalian toxicity compared to other types of insecticides [5]. However, recent studies have shown that pyrethroids may be associated with an increased risk of attention-deficit hyperactivity disorder, coronary heart disease, type 2 diabetes (T2D), delayed pubertal onset in children, negative impact on sperm DNA integrity and semen quality, and the possibility of cancers such as childhood brain tumors and acute lymphocytic leukemia [6,7,8,9,10,11,12]. Permethrin and cypermethrin are synthetic pyrethroids utilized for the management of a wide variety of home insect pests, such as mosquitoes, ants and cockroaches. In Thailand, cypermethrin ranked in the top 10 pesticides imported within the insecticide group in 2019–2020 [13,14]. In 2021, approximately 155 tons of cypermethrin were imported into Thailand [15]. Meanwhile, the Department of Industrial Work reported that 28 tons of permethrin were imported in 2021 [16].

There are only a few studies of children’s exposure to pyrethroids in Thailand. Previous studies reported pyrethroid exposure and household environment in children aged 6–8 years old [17], 1–3 years old [18], 2–3 years old [19]. As a result, there are gaps in our understanding of what factors (children’s characteristics, household characteristics and insecticide usage factors) may be associated with higher permethrin and cypermethrin exposure and metabolites in children. The objectives of this study are to improve our understanding of permethrin and cypermethrin exposures in young children aged 2–5 years old by measuring exposure levels on their hands and on home floor surfaces from insecticide usage and household characteristics; estimating health risks of children from wipe samples, as well as the urinary metabolite levels of the children; and identifying the factors associated with exposure to these pyrethroids in the residential environment.

## 2. Materials and Methods

### 2.1. Study Population and Data Collection

Young children aged 2 to 5 years and their parents or family caregivers were recruited from rural areas in Nakhon Pathom Province (Wang Nam Khiao sub-district) (*n* = 210) and Sing Buri provinces (Chi Nam Rai and Tha Ngam sub-districts) (*n* = 131), Thailand. The sub-district health-promoting hospitals and village health volunteers in the study areas helped to recruit the eligible participants and enroll the children and parents or family caregivers into the project. Finally, a total of 121 children participated in the study. The study was approved by the Committee on Human Rights Related to Human Experimentation, Faculty of Public Health, Mahidol University, Bangkok (MUPH 2019-093). All participants signed informed consent forms before participating in the study.

A questionnaire was employed to collect data on the socio-demographic information, housing characteristics, insecticide use within the household, and exposure behavior in their young children. Samples from floor surface wipes, children’s hands and urine were collected between September 2019 and May 2020. The researcher trained all staff in the method of collecting floor and hand wipe samples and how to interview parents and family caregivers. The parents or family caregivers were taught how to collect the morning void urine sample from their children, and the researcher and the trained staff provided the polyethylene bottle for urine collection to the parents or family caregivers the day before data collection. The data and urine collection were carried out at the home of the child.

Floor surface wipe samples were collected with a “4 × 4” cotton gauze pad, moistened with 70% isopropanol alcohol in an area of 30 cm × 30 cm template in the child’s main play area. The gauze pad was wiped in the designated area using an “S” shape from left to right, wiped top-to-bottom, and finally wiped around the perimeter. Both of the palms of the children’s hands were collected after parental assent using a “4 × 4” cotton gauze pad in the same way as surface wipes.

For urine sample collection, after the parent or family caregiver collected their children’s urine, the urine sample was frozen in their home refrigerator. The researcher came to collect the urine sample on the same day of urine collection. Urine samples were transported to the laboratory using a container with an ice pack and stored in a freezer at −20 °C until analysis.

### 2.2. Sample Analysis

#### 2.2.1. Chemical Reagents

For wipe sample analysis, permethrin was purchased from Dr. Ehrenstorfer, Augsburg, Germany (purity ≥ 99%). Cypermethrin and triphenyl phosphate were purchased from Sigma-Aldrich, Merck KGaA, Darmstadt, Germany. Acetonitrile (isocratic grade for liquid chromatography), toluene and isopropanol (analytical grade) were purchased from Supelco, Merck KGaA, Darmstadt, Germany. Primary and secondary amine was purchased from Agilent Technologies, Santa Clara, CA, USA, and magnesium sulfate anhydrous was purchased from Daejung, Gyeonggi-do, Korea. Methanol (for liquid chromatography grade) and acetic acid (reagent grade) were purchased from Merck KGaA, Darmstadt, Germany. For urinary metabolite analysis, 3-Phenoxybenzoic acid (3-PBA, purity ≥ 98%) and 2-Phenoxybenzoic acid (2-PBA, purity ≥ 98%), which were used as internal standards; tert-butyl methyl ether anhydrous (99.8%, analytical grade); and N,N’-Diisopropylcarbodiimide (DIC, 99% analytical grade) were obtained from Sigma-Aldrich, Merch KGaA, Darmstadt, Germany. cis-/trans-3-(2,2-dichlorovinyl)-2,2-dimethylcyclopropane carboxylic acid (cis/trans-DCCA, purity ≥ 98%) was purchased from Cambridge Isotope Laboratories, Inc., Andover, MA, USA. 1,1,1,3,3,3-Hexafluoro-2-propanol was analytical grade and obtained from ACROS Organics, Fair Lawn, NJ, USA. Acetonitrile (isocratic grade for liquid chromatography) and isooctane (analytical grade) were purchased from Supelco, Merck KGaA, Darmstadt, Germany. Hydrochloric acid fuming (37%) and sodium hydrogen carbonate were purchased from Merck KGaA, Darmstadt, Germany.

#### 2.2.2. Wipe Sample Analysis

All wipe samples were prepared using minor modifications of a protocol from a previous study [18]. Briefly, all wipe samples were extracted with acetonitrile, and triphenyl phosphate was used as an internal standard. The clear supernatant solution was evaporated at 40 °C to dryness under a gentle nitrogen stream and then reconstituted with toluene. Permethrin and cypermethrin were measured with a gas chromatography system 8890B (Agilent Technologies, USA) equipped with an autosampler and coupled to a 5977B mass selective detector (Agilent Technologies, USA). An Agilent HP-5ms capillary column (30 m × 0.25 mm i.d. ×0.25 µm film thickness) was used and the operating conditions were as follows: 80 °C held 2 min, then heated at a rate of 20 °C /min, ramped to 240 °C (20 min), followed by 50 °C /min, ramped to 290 °C held for 10 min, and finally post-run at 290 °C, held for 1 min. A sample volume was injected at 250 °C in splitless mode. Helium was used as carrier gas at a constant flow rate of 0.95 mL/min. The MSD transfer line, ion source and quadrupole temperatures were kept at 280, 230 and 150 °C, respectively. The monitored ions (*m*/*z*) for quantitation were 165, 183, 184 *m*/*z* for permethrin; 163, 165, 209 *m*/*z* for cypermethrin; and 168, 325, 326 *m*/*z* for triphenyl phosphate.

Calibration standard curves were prepared by spiking the standard solution of permethrin and cypermethrin onto each cotton gauze pad at concentrations of 0.025 to 1.5 µg and 1 to 25 µg. The accuracy of permethrin and cypermethrin ranged from 98.14 to 101.74% and 83.93 to 103.63% at concentrations of 0.1 and 1.0 µg, with a relative standard deviation (RSD) of less than 10. The limit of detection (LOD) was 0.010 µg for permethrin and 0.015 µg cypermethrin. The results from the hand wipe samples were reported as palmar hand loading (micrograms of pesticide per two hands of a child). The mass of permethrin and cypermethrin (µg) on the wipe samples was corrected using blanks and then divided by the wiped floor surface area (0.09 m^2^). The results of floor surface wipe samples were reported as µg/m^2^.

#### 2.2.3. Urine Sample Analysis

Urine samples were analyzed using the Leng and Gries method [20]. The three metabolites of permethrin and cypermethrin, consisting of 3-PBA, cis-DCCA and trans-DCCA, were measured using Gas Chromatography–Mass Spectrometry. Calibration standard curves were prepared by spiking the standard solution into blank urine at concentrations ranging from 0 to 150 ng/mL. The accuracy of cis-DCCA, trans-DCCA and 3-PBA ranged from 97.17 to 101.12%, 98.79 to 102.21 and 102.23 to 102.56%, respectively, at concentrations of 10 and 100 ng/mL with an RSD of less than 10. LOD was 0.20 ng/mL for cis-DCCA, trans-DCCA and 3-PBA, respectively. Creatinine measurements were analyzed to adjust the metabolite concentrations. The creatinine in urine was analyzed using an enzymatic method with a linear concentration range of 1–500 mg/dL and a detection limit of 0.16 mg/dL [21]. The units of metabolite concentrations were presented in nmoles/g creatinine. The results reported as cis- and trans-DCCA, 3-PBA and total pyrethroid metabolites calculated as the sum of cis- and trans-DCCA, and 3-PBA.

### 2.3. Health Risk Assessment of Hand Wipe Samples

The health risks of children from dermal contact with permethrin and cypermethrin were estimated by using hand wipe samples and presented in average daily dose (ADD), hazard quotient (HQ) and hazard index (HI) following the equation below [22,23].

(1) $$\mathrm{ADD}=\frac{{\mathrm{DA}}_{\mathrm{event}}\times \mathrm{EV}\times \mathrm{ED}\times \mathrm{EF}\times \mathrm{SA}}{\mathrm{BW}\times \mathrm{AT}}$$

where the abbreviations are as follows:

ADD = Average daily dose (mg/kg/day);
DA_event_ = Absorbed dose per event (mg/cm^2^/event); calculated from the concentration of a contaminant at 95th percentiles in hand wipe sample (mg) divided by skin surface area available for contact (SA) (cm^2^) and multiplied by absorption fraction (ABS) (unitless);
C = Concentration of contaminant at 95th percentiles in hand wipe sample (mg);
SA = Skin surface area available for contact (cm^2^);
ABS = Absorption fraction (unitless);
EF = Exposure frequency (days/year);
EV = Event frequency (events/days);
ED= Exposure duration (year): average age of children based on questionnaire results;
BW = Body weight (kg): average based on questionnaire results;
AT = Average time (days): ED × 365 days.

Hazard quotient (HQ) = ADD/RfD(2)

where the abbreviations are as follows:

HQ = Hazard quotient (unitless);
ADD = Average daily dose (mg/kg/day);
RfD = Reference dose (mg/kg/day) for permethrin = 0.05 [24], cypermethrin 0.01 mg/kg/day [25].

A hazard quotient less than or equal to 1 indicates that adverse non-carcinogenic effects are not likely to occur and thus can be considered to have negligible hazard. If a hazard quotient greater than 1 indicates that adverse non-carcinogenic effects are a concern.

(3) Hazard index (HI)=∑HQ

The hazard index (HI) is the sum of hazard quotients for multiple substances. If the Hazard Quotient is less than 1, no adverse health effects are expected from exposure. However, if the Hazard Quotient is greater than 1, there is a possibility of adverse health effects.

### 2.4. Statistical Analysis

All data analyses were performed using SPSS software for Windows, version 23 (IBM Thailand Co., Ltd., Bangkok, Thailand). Descriptive statistics were used to summarize and describe permethrin, cypermethrin wipe levels and their urinary metabolite concentrations. For samples where the concentration of the analytes fell below the LOD, the LOD divided by 2 was used [26]. Urine samples with creatinine levels out of the range of 30 to 300 mg/dL were excluded from these analyses [27,28,29]. Urinary creatinine concentrations of the children in this study ranged from 0.50 to 224 mg/dl. Therefore, the statistical analysis of urine samples in this study included 99 children, with 22 urine samples excluded. As a rule of thumb, urine results with a z score greater than 3 or less than −3 are often determined to be outliers [30]. Furthermore, two urine samples that were outliers were identified, and these were excluded from the data analysis. In the end, a total of 97 urine samples were analyzed. The urinary metabolite concentrations were converted into the natural logarithm for statistical analysis after the results showed that the data were highly skewed and not normally distributed. The Wilcoxon signed-rank test was used to compare the difference between permethrin and cypermethrin concentrations for the wipe samples due to the high percentage of LODs and/or the very large GSDs. The Mann–Whitney U test and Kruskal–Wallis test were used to compare the concentrations of these wipe samples in relation to various factors. Meanwhile, independent *t*-tests and one-way ANOVA were used to compare the concentrations of the urine samples.

The factors, including all variables for children characteristics, household characteristics and pyrethroid usage in the household except for insecticide storage areas and exposure-related behavior in children (Table 1) associated with hand wipe and floor wipe samples of permethrin and cypermethrin were first analyzed using a univariate generalized linear model (GLM). Variable with *p*-value ≤ 0.100 are reported. Next, we adjusted the models so that they included baseline covariates that could influence wipe levels, such as age and children’s gender [18], having family members involved in agriculture (significant in this study) and distance from home to agricultural area [31,32]. Then, we re-ran the single variable GLM models. Lastly, we developed a stepwise multivariable regression model for hand wipe and floor wipe samples of permethrin and cypermethrin using parameters with *p*-value ≤0.100 and including the baseline covariates. We checked the correlation of all parameters before putting them into the multivariable model; we found a significant correlation between permethrin in children’s hands and surface floors and cypermethrin in children’s hands and surface floors.

For permethrin and cypermethrin in floor surfaces of the child’s main play area, the baseline covariates used were having family members involved in agriculture and distance from home to agricultural area only.

The factors associated with urinary total pyrethroid metabolites, 3-PBA, and cis- and trans-DCCA were also analyzed using this 3-step procedure. Since Ln permethrin and cypermethrin on children’s hands and surface floors were significantly correlated, they could not be put into the same multivariable model. We created four models by putting one of these variables in each of the models as models 1–4. The model that was not significant was excluded.

## 3. Results

### 3.1. General Demographic Characteristics

Of the 121 children in this study, 52.9% were boys, and 47.1% were girls (Table 1). The median age of the children was 4.00 years (IQR = 3.00–5.00). A total of 53.7% of children had family members involved in agriculture, and the average distance from home to the agricultural area was 581.99 m (SD = 2005.44). The floor types were tile (47.1%), cement (46.3%) and wood (6.6%). The percentage of participants who cleaned their houses every day was 69.4%, and not every day per week was 30.6%. A total of 80.2% always opened the windows of their house every day, and 18.2% opened them sometimes. A total of 75.2% had used household insecticide products in their residential area within the past three months. Among them, 56.2% used aerosol insecticide sprays, 24.8% used mosquito coils, 9.1% used both and 5.8% used other methods such as chalk. For those who used household insecticide products, 54.5% reported mainly using them in the living room, 41.3% in the bedroom and 28.9% in the kitchen. Moreover, they mostly stored the insecticides at home (88.4%) in the living room (42.1%), terrace/patio (27.3%) and others such as the kitchen (19.0%). For exposure-related behaviors of children, 75.2% played inside, and 14.1% played outside the house during the daytime. A total of 60.3% of them spent the most time in the living room or bedroom, while 39.7% spent time on the terrace/patio.

### 3.2. Permethrin and Cypermethrin Concentrations in Children’s Hand, Floor Surface Wipes and the Urinary Pyrethroid Metabolites of the Children

Permethrin and cypermethrin concentrations on the children’s hand and floor surface wipe samples are presented in the units of µg (per palmar surface of both hands) and µg/m^2^, respectively, as shown in Table 2. In children’s hand wipe samples, cypermethrin (82.64%) was detected higher than permethrin (61.98%). In surface floor wipe samples, cypermethrin (92.56%) was also detected higher than permethrin (79.34%). The median of cypermethrin (0.04 µg) was significantly higher than permethrin (0.02 µg) in children’s hand wipe samples. The median of cypermethrin (1.38 µg/m^2^) was not significantly different from permethrin (1.02 µg/m^2^) in the floor surface wipe samples. The detection frequency of urinary pyrethroid metabolites for children was highest for 3-PBA (89.7%), followed by cis- and trans-DCCA (86.6%).

### 3.3. Comparison of Permethrin Concentrations on the Children’s Hands and Floor Surfaces

The permethrin and cypermethrin concentrations on the children’s hands and floor surfaces were not significantly different for boys and girls (Table 3). Meanwhile, children who had family members involved in agriculture had significantly lower permethrin concentrations on the children’s hands and floor surfaces than those who did not. Houses with tile floors had significantly higher permethrin on the floor surfaces than those with cement and wood floors. The families who reported opening the windows of their homes had significantly lower permethrin on their children’s hands and floor surfaces than those who did not. Households using insecticide products in the residential area within the past 3 months had significantly higher permethrin levels (1.64 µg/m^2^) on the floor surface than those who did not (0.23 µg/m^2^). The permethrin concentrations on the children’s hands and floor surfaces were significantly higher in the households that used aerosol insecticide sprays than those that did not. In contrast, the families who used mosquito coils had significantly lower permethrin on their children’s hands and surface floors than those who did not. The permethrin concentration on the children’s hands was significantly higher in families using insecticides in the living room and bedroom compared to those who did not. Similar results were observed for the floor surface wipes, where homes that reported using insecticides in the living room and bedroom had significantly higher permethrin levels. The families who stored insecticides at home had significantly higher permethrin on their children’s hands and floor surfaces than those who did not.

### 3.4. Comparison of Cypermethrin Concentrations on the Children’s Hands and Floor Surfaces

The cypermethrin concentrations on the children’s hands and floor surface wipes were affected by household characteristics, especially types of floors, but they were not affected by either children’s characteristics or insecticide usage in residential areas (Table 3). 

### 3.5. Comparison of the Urinary Pyrethroid Metabolite Concentrations in Children

The urinary metabolite concentrations were not significantly different based on children’s gender or having family members involved in agriculture (Table 4). Families who used tile as the common floor type had significantly higher 3-PBA compared to those who did not. There were no significant differences in urinary pyrethroid metabolite concentrations in children found for household insecticide product usage.

### 3.6. Factors Impacting Permethrin Concentrations on Children’s Hands and Floor Surfaces

In the multivariable model, the families who reported using mosquito coils and insecticide in the living room were the most important predictors of permethrin concentrations on the children’s hands (Table 5). The families who always or sometimes opened the window had significantly lower permethrin concentrations on floor surfaces, but those using the household insecticide product within the past 3 months and aerosol insecticide sprays had significantly higher permethrin concentrations on floor surfaces (Table 6).

### 3.7. Factors Impacting of Cypermethrin Concentrations on the Children’s Hands and Floor Surfaces

Table 7 reports only those variables whose *p*-value was ≤0.100 in crude models of floor surfaces of the child main’s play areas. However, cypermethrin concentrations on the floor surface of the child’s main play area were significantly higher in families using aerosol insecticide sprays, and household insecticide products in the living room and bedroom compared to those who did not (Table 7). In the multivariable model, the most important predictors of cypermethrin concentrations on the children’s hands and floor surfaces of the children’s main play area are not found.

### 3.8. Factors Impacting the Urinary Pyrethroid Metabolite Concentrations in Children

An increase in one nmole/g creatinine of Ln total pyrethroid metabolites significantly increased Ln permethrin in children’s hand for 1.38 µg, Ln permethrin in floor surface for 1.28 µg/m^2^ and Ln cypermethrin in floor surface for 1.32 µg/m^2^ after controlling for age, gender, having family members involved in agriculture, distance from home to agricultural area (Table 8). The increase in one nmole/g creatinine of Ln DCCA significantly increased Ln permethrin in children’s hand for 1.32 µg, Ln permethrin in floor surface for 1.22 µg/m^2^ and Ln cypermethrin in floor surface for 1.28 µg /m^2^ after controlling for age, gender, having family members involved in agriculture, distance from home to agricultural area (Table 9). The most important predictors of the total pyrethroid and DCCA metabolites are permethrin on children’s hands or floor surfaces and cypermethrin on floor surfaces.

Other predictors of urinary metabolite levels were less consistent. With 3-PBA, tile floors and the use of aerosol insecticide sprays significantly increased 3-PBA in the children’s urine, even after controlling for age, gender, family members involved in agriculture, and distance from home to agricultural area (Table 10). In a multivariable model, the most important predictors of 3-PBA are tile floors, permethrin and cypermethrin on children’s hands or floor surfaces.

### 3.9. Health Risk Assessment of Hand Wipe Samples

The health risk assessment from dermal exposure to cypermethrin in the children’s hands in this study is presented in Table 11. The results demonstrated that the ADD from dermal exposure to permethrin and cypermethrin were 5.68 × 10^−7^ mg/kg/day and 1.63 × 10^−7^ mg/kg/day, respectively. The hazard quotient of both permethrin and cypermethrin was below 1. Moreover, the hazard index was below 1, which indicated that dermal exposure to permethrin and cypermethrin in this study was negligible.

## 4. Discussion

The findings of the present study indicate that young children are exposed to permethrin and cypermethrin within their residential environment, as previous studies have reported [17,18,19,37]. Our permethrin floor surface concentrations were higher than those of Trunnelle et al. (0.174 µg/m^2^) but lower than those of Stout et al. (2.4 µg/m^2^), both of whom were in the U.S. [38,39]. Floor wipes concentrations may have varied due to household cleanliness [38], time since last usage or type of insecticide usage/storage or application pattern. The cypermethrin floor surface concentrations in this current study were lower than those of Siriwat et al. (4.13 µg/m^2^) during the wet season in Thailand because Siriwat et al. (2019) collected samples from children living in areas where farmers used pesticides intensively [18]. In addition, Siriwat et al. (2019) reported that cypermethrin concentrations were higher in the wet season due to the increased presence of insects inside the house during this period compared to the dry season [18]. The cypermethrin floor surface concentrations in this current study were higher than the study of Stout et al. (0.016 µg/m^2^) from the U.S., probably because our Thai family used insecticides more frequently in residential areas [19].

For children’s hand wipe samples, the study of Siriwat et al. (2019) reported a higher detection frequency of cypermethrin in all children’s hand and floor wipe samples (100%). Meanwhile, the average concentration of cypermethrin on children’s hands of Siriwat et al. (2019) was 0.096 µg during the dry season and 0.503 µg during the wet season [18]. In this study, we only collected samples during the dry season and could not account for seasons in our analyses. Previous studies have reported that partially opening the windows can decrease the persistence of permethrin and reduce pyrethroid concentrations in indoor air [40,41]. Floor surface samples showed that tile floors had significantly higher permethrin concentrations than other flooring materials. This may be due to lower porosity and roughness of tile floors than cement or wood floors. A previous study revealed that tile had a higher overall pesticide collection efficiency than hardwood [42]. 

Among families who reported using insecticides in the past three months, children’s hand and floor surface wipes for permethrin were significantly higher than those who had not used insecticides recently. This may be because permethrin is readily adsorbed by surfaces, and the concentration declines by 10% in 12 months [43] or 40% in 112 days [44]. However, cypermethrin residues can last for 84 days in the air, on walls, the floor and on furniture [45]. Cypermethrin is used both on the farm and in the home [37], but permethrin is used mainly in the home for insect control. In our study area, aerosol insecticide sprays were the main type of household insecticide product used. Permethrin was reported as the main active ingredient more often than cypermethrin in the aerosol insecticide sprays used in Thailand [46,47]. However, the use of household cleansers can lead to a decrease in the concentration levels of residual pyrethroids on indoor surfaces [43].

Those who reported using aerosol insecticide sprays had significantly higher permethrin in hands and floor surface concentrations, while those who reported using mosquito coils had significantly lower permethrin concentrations on their children’s hands and floor surfaces. This may be explained by the difference in their active ingredients; the main active ingredient of aerosol insecticide sprays contained permethrin, cypermethrin and other pyrethroids, whereas mosquito coils often contained other pyrethroids such as d-allethrin, metrofluthrin, esbiothrin, meperfluthrin and transfluthrin, but less frequently permethrin and cypermethrin [47,48]. Families who reported using household insecticide products in the living room or bedroom, as well as those who reported storing insecticides at home, had significantly higher permethrin on their children’s hands and floor surfaces. In this study, the children mostly stayed in the living room and bedroom during the daytime. A previous study showed that floor concentrations were significantly associated with the location where the insecticide was applied (living room and bedroom) or the place where the insecticide was stored [49]. 

The GM concentrations of 3-PBA and DCCA were 1.47 and 4.01 µg/g creatinine, respectively. The urinary 3-PBA concentration was 1.47 µg/g creatinine in this study, 1.71 µg/g creatinine in Japan [50], 1.1 µg/g creatinine in Australia [51] and 2.24 µg/g creatinine [17] in the dry season in Thailand. The variation in reported 3-PBA concentrations may be due to different use patterns in various regions or because 3-PBA is a non-specific pyrethroid metabolite and may reflect exposure to other compounds. The DCCA concentration was higher (4.01 µg/g creatinine) in this study compared to 1.13 µg/g creatinine in Japan [50], 3.6 µg/g creatinine in Australia [51] and 1.11 µg/g creatinine in Thailand [17]. This may be because our study was rural, and Babina et al. reported that children who lived within 50 m or less of an agricultural activity had much higher urinary DCCA levels due to exposure to permethrin, cypermethrin and cyfluthrin, which are all frequently used in agriculture and available for household use [51].

Although food consumption is regarded as the main route of pyrethroid exposure in the general population [52,53,54], the home use of insecticides is also an important route [55,56,57]. The lack of association between urinary metabolite levels and household insecticide product usage characteristics may be partly because of the short half-lives of these metabolites. The urinary permethrin and cypermethrin metabolites have very short half-lives in human urine, 3-PBA (5.7 h) and DCCA (4.5–5.4 h) [58]. The half-life of their metabolites was 12.3 h in serum and 9–23 h in tissue and brain [59]. The primary part could be from children’s consumption of food and vegetables contaminated with pesticides in their everyday lives, which is continuous exposure. The detection of permethrin and cypermethrin residues was found in vegetables and fruits in Thailand’s markets. Prapamontol et al. (2020) reported that cypermethrin (21.6%) and permethrin (2.9%) residues exceeded the maximum residue limit for pesticides (MRL) in vegetable and fruit samples sourced from markets in urban and rural areas of upper northern Thailand during 2007–2013 [60]. Suntudrob et al. (2018) also reported that vegetables, including Chinese broccoli, ivy gourd, yard long bean and water spinach from wholesale markets in 77 provinces contained detectable pesticide residues ranging from <0.01 to 5.9 mg/kg [61]. The results showed that children may be exposed to permethrin and cypermethrin through the consumption of these vegetables and fruits. Another reason for the lack of association between urinary metabolite levels and household insecticide product usage could be explained by the administration technique of insecticide sprays. The insecticide was used by spraying insecticide in the air (aerosol spray), burning the mosquito coil in the air (mosquito coil) and drawing the chalk line (chalk); the aerosol would be less adsorbed onto the children’s hands.

There was an association between total pyrethroid metabolites, DCCA, 3-PBA, and permethrin and cypermethrin concentrations on both children’s hands and floor surfaces. These results are consistent with previous studies. Tulve et al. (2008) found a significant correlation between cypermethrin levels in the floor wipes of application areas and play areas, as well as the metabolites of DCCA and 3-PBA. Trunnelle et al. (2014) reported that floor wipe pyrethroid concentrations were correlated with urinary concentrations of 3-PBA in children. Rohitrattana et al. (2014) found a positive correlation between cypermethrin concentrations on children’s hands and pyrethroid metabolites in children’s urine. Kunno et al. (2020) reported a positive correlation between 3-PBA and cypermethrin concentrations on children’s hand wipes [2,17,19,38]. These correlations may be due to the fact that many household insecticide products mainly contain permethrin, cypermethrin and other pyrethroids as active ingredients [46,47]. 

The health risk assessment results of this study on dermal contact with permethrin and cypermethrin are consistent with the findings of Siriwat et al. (2019) [18], which demonstrate that the risk of dermal exposure to household insecticides is very low and not a cause for concern.

This study did not collect samples during different seasons, which may play an important role in affecting household insecticide concentration and the frequency of detection. Moreover, this study had a limited sample size, which could impact the evaluation of exposure determinants. This study collected hand wipe and floor surface wipe samples only once in the dry season and only one first morning void urine sample from the children during that time. However, the use of insecticides in the home varies by season in Thailand and could result in different findings.

## 5. Conclusions

The current study evaluated the residential exposure to permethrin and cypermethrin among young children ages 2 to 5 years. This study identified higher permethrin concentrations on children’s hands and floor surfaces in homes that recently used household insecticide products, especially aerosol insecticide sprays, or applied insecticides in the living room and bedroom or stored them at home. Tile floors and window openings, as household characteristics, were also associated with varying permethrin concentrations. Furthermore, higher cypermethrin concentrations on floor surfaces were observed in homes using aerosol insecticide sprays and applying insecticide products in the living room and bedroom. For urinary metabolites, this study found significant associations between permethrin concentrations on the children’s hands and floor surfaces and cypermethrin on floor surfaces and total pyrethroid metabolites, DCCA and 3-PBA. In addition, 3-PBA was associated with using aerosol insecticide sprays, tile floors at home and cypermethrin on children’s hands. The health risk assessment of hand wipe samples indicated that dermal exposure to permethrin and cypermethrin was negligible. This study recommends that family caregivers clean the floor before performing any activities in those areas after using household insecticide products and store insecticides outside the house to reduce the insecticide exposure of young children in the residential environment.

## Figures and Tables

**Table 1:** General demographic characteristics (*n* = 121).

Variables	*n* (%)
Demographics	
Children’s gender	
Boy	64 (52.9)
Girl	57 (47.1)
Children’s age (years)	
Median (IQR)	4.00 (3.00–5.00)
Having family members involved in agriculture	
Yes	65 (53.7)
No	56 (46.3)
Household characteristic	
Distance from home to the agricultural area (meters)	
Mean (SD)	581.99 (2005.44)
Floor types	
Cement	56 (46.3)
Wood	8 (6.6)
Tile	57 (47.1)
Cleaning house every day	
Yes	84 (69.4)
No	37 (30.6)
Opening the window	
Always	97 (80.2)
Sometimes	22 (18.2)
Never	2 (1.7)
Insecticide usage	
Using the household insecticide product in the residential area within the past 3 months	
Yes	91 (75.2)
No	30 (24.8)
Type of household insecticide products	
Aerosol insecticide sprays	68 (56.2)
Mosquito coils	30 (24.8)
More than 2 types	11 (9.1)
Others (ex. chalk)	7 (5.8)
Do not use	5 (4.1)
Insecticide use area (multiple choices)	
Living room	66 (54.5)
Kitchen	35 (28.9)
Bedroom	50 (41.3)
Insecticide storage at home	
Yes	107 (88.4)
No	14 (11.6)
Insecticide storage area	
Living room	51 (42.1)
Terrace/Patio	33 (27.3)
Others (ex. kitchen)	23 (19.0)
Not Stored	14 (11.6)
Exposure-related behavior in children	
Area where the child spends the longest time at home during the day	
Living room/bedroom	73 (60.3)
Terrace or patio	48 (39.7)
Activity that the child does most during the daytime	
Playing inside the house	91 (75.2)
Playing outside the house	17 (14.1)
Other	13 (10.7)

**Table 2:** Permethrin and cypermethrin concentrations on children’s hand wipes, floor surface wipes and the children’s urinary pyrethroid metabolite concentrations.

Concentrations	Detection Frequency (%)	GM (GSD) ^1^	Median	P25–P75 ^2^	*p*-Value ^3^
Children’s hand wipe samples (µg) (*n* = 121)					
Permethrin	75 (61.98)	0.02 (3.69)	0.02	0.01–0.04	<0.001 ***
Cypermethrin	100 (82.64)	0.04 (3.26)	0.04	0.02–0.08	
Floor surface wipe samples (µg/m^2^) (*n* = 121)					
Permethrin	96 (79.34)	0.90 (8.28)	1.02	0.17–3.03	0.157
Cypermethrin	112 (92.56)	1.49 (4.86)	1.38	0.45–4.61	
Urinary pyrethroid metabolites (nmol/g Creatinine) (*n* = 97)					
cis- and trans-DCCA	84 (86.6)	0.84 (2.64)	1.00	0.50–1.71	
3-PBA	87 (89.7)	0.31 (2.98)	0.38	0.18–0.60	
Total pyrethroid metabolite		1.23 (2.47)	1.41	0.79–2.20	

^1^ GM refers to geometric mean, and GSD refers to geometric standard deviation. ^2^ P25 and P75 refer to 25 percentiles and 75 percentiles. ^3^ *p*-value were calculated using a Wilcoxon signed-rank test, *** ≤ 0.001. LOD = 0.010 µg per sample for permethrin, 0.015 µg per sample for cypermethrin in wipe samples; LOD = 0.20 ng/mL for cis- and trans-DCCA, and 3-PBA in urine samples.

**Table 3:** Comparison of permethrin and cypermethrin concentrations on the children’s hands and floor surfaces (*n* = 121).

Parameter		Median (IQR) ^1^			
		Children’s Hands (µg)		Floor Surface (µg/m^2^)	
		Permethrin	Cypermethrin	Permethrin	Cypermethrin
Demographics					
Children’s gender					
	Boy	0.02 (<LOD–0.04)	0.04 (0.02–0.08)	0.99 (0.15–2.66)	1.72 (0.54–4.79)
	Girl	0.02 (<LOD–0.05)	0.04 (0.02–0.07)	1.07 (0.17–3.99)	1.27 (0.42–4.61)
	*p*-value ^2^	0.940	0.492	0.876	0.550
Having family members involved in agriculture					
	Yes	<LOD (<LOD–0.03)	0.04 (0.02–0.07)	0.44 (0.06–2.21)	1.43 (0.39–4.96)
	No	0.02 (0.01–0.05)	0.03 (0.02–0.08)	2.09 (0.31–4.72)	1.32 (0.54–4.02)
	*p*-value ^2^	0.002 **	0.613	0.014 *	0.905
Household characteristics					
Cleaning house every day					
	Yes	0.02 (<LOD–0.05)	0.03 (0.02–0.07)	1.06 (0.19–2.84)	1.29 (0.44–4.38)
	No	0.01 (<LOD–0.05)	0.05 (0.02–0.08)	0.90 (0.11–3.64)	2.65 (0.45–5.10)
	*p*-value ^2^	0.623	0.286	0.747	0.532
Floor types					
	Cement (C)	0.01 (<LOD–0.03)	0.05 (0.02–0.1)	0.62 (0.19–2.57)	1.37 (0.63–3.95)
	Wood (W)	<LOD (<LOD–0.05)	0.02 (<LOD–0.06)	0.06 (0.06–1.03)	0.23 (0.11–0.51)
	Tile (T)	0.02 (<LOD–0.05)	0.04 (0.02–0.08)	1.90 (0.29–3.91)	1.72 (0.44–6.11)
	*p*-value				
	(C)-(W) ^3^	0.696	0.044 *	0.038 *	0.003 **
	(C)-(T) ^3^	0.0866	0.565	0.187	0.497
	(W)-(T) ^3^	0.263	0.068	0.006 *	< 0.001 ***
	(C)-(W)-(T) ^4^	0.171	0.131	0.019 *	0.004 **
Opening the window					
	Always or sometimes	0.02 (<LOD–0.04)	0.04 (0.02–0.07)	0.97 (0.17–2.90)	1.35 (0.45–4.57)
	Never	0.20 (0.15)	0.05 (0.03)	152.03 (91.12)	7.27 (3.87)
	*p*-value ^2^	0.012 *	0.702	0.002 **	0.172
Insecticide usage					
Using the household insecticide product in the residential area within the past 3 months					
	Yes	0.02 (<LOD–0.05)	0.04 (0.02–0.08)	1.64 (0.30–3.99)	1.53 (0.64–4.91)
	No	<LOD (<LOD–0.03)	0.04 (<LOD–0.07)	0.23 (0.06–1.38)	0.70 (0.33–3.96)
	*p*-value ^2^	0.030 *	0.551	< 0.001 ***	0.110
Aerosol insecticide sprays					
	Yes	0.02 (<LOD–0.06)	0.04 (0.02–0.07)	1.99 (0.33–5.06)	1.73 (0.46–5.08)
	No	0.01 (<LOD–0.02)	0.05 (0.02–0.08)	0.26 (0.06–0.93)	0.85 (0.35–3.10)
	*p*-value ^2^	0.011 *	0.528	< 0.001 ***	0.065
Mosquito coil					
	Yes	0.01 (<LOD–0.02)	0.05 (0.02–0.09)	0.31 (0.11–1.47)	0.83 (0.34–3.57)
	No	0.02 (0.01–0.05)	0.04 (0.02–0.08)	1.90 (0.30–4.16)	1.72 (0.46–5.29)
	*p*-value ^2^	0.047 *	0.264	0.004 **	0.086
Insecticide use in living room					
	Yes	0.02 (<LOD–0.06)	0.04 (0.02–0.10)	1.66 (0.32–3.39)	1.72 (0.66–6.06)
	No	0.01 (<LOD–0.02)	0.03 (0.02–0.06)	0.37 (0.06–2.34)	1.28 (0.4–3.97)
	*p*-value ^2^	0.004 **	0.175	0.009 **	0.086
Insecticide use in bedroom					
	Yes	0.02 (<LOD–0.06)	0.04 (0.02–0.08)	1.89 (0.30–4.03)	2.75 (0.59–6.60)
	No	0.01 (<LOD–0.03)	0.04 (0.02–0.07)	0.50 (0.06–2.61)	0.99 (0.42–3.97)
	*p*-value ^2^	0.052	0.922	0.044 *	0.065
Store insecticide at home					
	Yes	0.02 (<LOD–0.05)	0.04 (0.02–0.08)	1.38 (0.22–3.82)	1.53 (0.46–4.89)
	No	<LOD (<LOD–0.02)	0.03 (<LOD–0.06)	0.23 (0.06–0.63)	1.05 (0.23–2.78)
	*p*-value ^2^	0.021 *	0.202	0.003 **	0.173

^1^ IQR refers to the interquartile range. *p*-value was calculated using ^2^ Mann–Whitney U test, ^3^ Independent sample pairwise comparisons, ^4^ Kruskal–Wallis test; * ≤ 0.05, ** ≤ 0.01, *** ≤ 0.001.

**Table 4:** Comparison of permethrin and cypermethrin urinary metabolite concentrations classified by the demographics, household characteristics and household insecticide product usage (*n*= 97).

Parameter		GM (GSD) ^1^; nmol/g Creatinine		
		Cis- and Trans-DCCA	3-PBA	Total Pyrethroid Metabolite
Demographics				
Children’s gender				
	Boy	0.75 (2.80)	0.28 (3.37)	1.10 (2.63)
	Girl	0.97 (2.43)	0.36 (2.50)	1.40 (2.28)
	*p*-value ^2^	0.192	0.235	0.204
Having family members involved in agriculture				
	Yes	0.97 (2.43)	0.31 (2.83)	1.16 (2.47)
	No	0.92 (2.62)	0.32 (3.18)	1.31 (2.50)
	*p*-value ^2^	0.381	0.924	0.515
Household characteristics				
Cleaning house every day				
	Yes	0.77 (2.83)	0.29 (3.23)	1.13 (2.68)
	No	1.04 (2.17)	0.37 (2.39)	1.51 (1.96)
	*p*-value ^2^	0.153	0.291	0.142
Floor types				
	Cement (C)	0.84 (2.65)	0.27 (3.03)	1.19 (2.45)
	Wood (W)	0.48 (3.62)	0.11 (4.52)	0.66 (3.42)
	Tile (T)	0.89 (2.55)	0.41 (2.55)	1.36 (2.39)
	*p*-value			
	(C)-(W) ^3^	0.219	0.075	0.164
	(C)-(T) ^3^	0.780	0.049 *	0.484
	(W)-(T) ^3^	0.176	0.009 **	0.090
	(C)-(W)-(T) ^4^	0.399	0.012 *	0.233
Opening the window				
	Always or sometimes	0.84 (2.67)	0.31 (3.01)	1.23 (2.50)
	Never	1.08 (1.08)	0.39 (1.44)	1.48 (1.04)
	*p*-value ^2^	0.793	0.782	0.770
Insecticide usage				
Using insecticide in the residential area within the past 3 months				
	Yes	0.87 (2.79)	0.34 (2.92)	1.29 (2.60)
	No	0.76 (2.24)	0.24 (3.08)	1.09 (2.11)
	*p*-value ^2^	0.558	0.156	0.431
Aerosol insecticide sprays				
	Yes	0.90 (2.78)	0.36 (2.86)	1.32 (2.61)
	No	0.73 (2.33)	0.23 (3.07)	1.06 (2.16)
	*p*-value ^2^	0.334	0.051	0.251
Mosquito coil				
	Yes	0.85 (2.82)	0.29 (3.00)	1.21 (2.65)
	No	0.84 (2.58)	0.32 (2.99)	1.24 (2.40)
	*p*-value ^2^	0.917	0.636	0.912
Insecticide use in living room				
	Yes	0.96 (2.72)	0.34 (3.26)	1.40 (2.50)
	No	0.74 (2.54)	0.29 (2.70)	1.08 (2.43)
	*p*-value ^2^	0.187	0.479	0.161
Insecticide use in bedroom				
	Yes	0.94 (2.93)	0.39 (3.13)	1.37 (2.88)
	No	0.78 (2.45)	0.27 (2.81)	1.14 (2.19)
	*p*-value ^2^	0.372	0.085	0.344
Store insecticide at home				
	Yes	0.90 (2.70)	0.33 (2.97)	1.31 (2.51)
	No	0.54 (1.94)	0.20 (2.85)	0.80 (1.94)
	*p*-value ^2^	0.091	0.132	0.074

^1^ GM refers to geometric mean, and GSD refers to geometric standard deviation. *p*-values were calculated using ^2^ independent *t*-test, ^3^ multiple comparison with LSD, ^4^ one-way ANOVA; * ≤ 0.05, ** ≤ 0.01.

**Table 5:** Generalized linear models (GLM) of natural log (Ln) permethrin on children’s hands (µg) with one parameter for household characteristics or insecticide usage (crude models). Adjusted models also include covariates for age, gender, family members involved in agriculture, and distance from home to agricultural area, in addition to the single parameter for household characteristics or insecticide usage (adjusted model). A multivariable model including age, gender, family members involved in agriculture, and distance from home to agricultural area is also presented (*n* = 121).

Parameters	GLM				Multivariable Model	
	Crude OR ^1^ (95% CI ^2^)	*p*-Value	Adjusted OR ^1^ (95% CI ^2^)	*p*-Value	Adjusted OR ^1^ (95% CI ^2^)	*p*-Value
Tile floor (1/0) ^3^	1.61 (1.02–2.54)	0.041 *	1.66 (1.06–2.60)	0.027 *		
Always or sometime opening the window (1/0) ^3^	0.09 (0.02–0.53)	0.007 **	0.12 (0.02–0.69)	0.017 *		
Using the household insecticide product in the residential area within the past 3 months (1/0) ^3^	1.83 (1.08–3.09)	0.024 *	1.49 (0.87–2.55)	0.148		
Aerosol insecticide sprays (1/0) ^3^	2.02 (1.25–3.26)	0.004 **	2.16 (1.34–3.46)	0.001 ***		
Mosquito coils (1/0) ^3^	0.57 (0.35–0.91)	0.020 *	0.57 (0.36–0.90)	0.017 *	0.78 (0.33–0.81)	0.004 **
Insecticide use in living room (1/0) ^3^	1.98 (1.27–3.11)	0.003 **	2.03 (1.32–3.14)	0.001 ***	1.34 (1.40–3.31)	0.001 ***
Insecticide use in bedroom (1/0) ^3^	1.82 (1.15–2.87)	0.011 *	1.75 (1.12–2.73)	0.014 *		
Insecticide storage at home (1/0) ^3^	2.29 (1.13–4.66)	0.022 *	2.24 (1.12–4.48)	0.022 *		

^1^ OR refers to odds ratio. ^2^ CI refers to confidence interval. ^3^ Reference category: 1 = yes; 0 = no. *p*-value were calculated using a GLMs and multivariable model; * ≤ 0.05, ** ≤ 0.01, *** ≤ 0.001.

**Table 6:** GLM of Ln permethrin on floor surfaces of child’s main play area (µg/m^2^) with one parameter for household characteristics or insecticide usage. Adjusted models also include covariates for having family members involved in agriculture, and distance from home to agricultural area, in addition to the single parameter for household characteristics or insecticide usage. A multivariable model, including family members involved in agriculture, and distance from home to agricultural area, is also presented (*n* =121).

Parameters	GLM				Multivariable Model	
	Crude OR ^1^ (95% CI ^2^)	*p*-Value	Adjusted OR ^1^ (95% CI ^2^)	*p*-Value	Adjusted OR ^1^ (95% CI ^2^)	*p*-Value
Tile floor (1/0) ^3^	2.05 (0.96–4.29)	0.058	2.10 (1.01–4.39)	0.048 *		
Always or sometimes opening the window (1/0) ^3^	0.01 (0.00–0.10)	<0.001 ***	0.01 (0.00–0.14)	<0.001 ***	0.77 (0.00–0.17)	0.001 ***
Using the household insecticide product in the residential area within the past 3 months (1/0) ^3^	4.10 (1.78–9.41)	0.001 ***	3.35 (1.41–7.96)	0.006 **	1.24 (1.30–6.23)	0.009 **
Aerosol insecticide sprays (1/0) ^3^	5.30 (2.52–11.18)	<0.001 ***	5.64 (2.73–11.67)	<0.001 ***	1.36 (1.95–8.27)	<0.001 ***
Mosquito coils (1/0) ^3^	0.31 (0.15–0.67)	0.003 **	0.31 (0.15–0.66)	0.002 **		
Insecticide use in living room (1/0) ^3^	2.70 (1.30–5.61)	0.008 **	2.74 (1.34–5.59)	0.006 **		
Insecticide use in bedroom (1/0) ^3^	2.28 (1.08–4.82)	0.031 *	2.18 (1.04–4.55)	0.038 *		
Insecticide storage at home (1/0) ^3^	5.47 (1.76–17.00)	0.003 **	5.28 (1.73–16.11)	0.003 **		

^1^ OR refers to odds ratio.^2^ CI refers to confidence interval. ^3^ Reference category: 1 = yes; 0 = no. *p*-value were calculated using a GLMs and multivariable model; * ≤ 0.05, ** ≤ 0.01, *** ≤ 0.001.

**Table 7:** GLM of Ln cypermethrin on floor surfaces of child’s main play area (µg/m^2^) with one parameter for household characteristics or insecticide usage. Adjusted models also include covariates for having family members involved in agriculture, and distance from home to the agricultural area, in addition to the single parameter for household characteristics or insecticide usage (*n* = 121).

Parameters	GLM			
	Crude OR ^1^ (95% CI ^2^)	*p*-Value	Adjusted OR ^1^ (95% CI ^2^)	*p*-Value
Aerosol insecticide sprays (1/0) ^3^	1.71 (0.95–3.09)	0.076	1.79 (0.99–3.24)	0.053
Insecticide use in living room (1/0) ^3^	1.76 (1.01–3.06)	0.046 *	1.77 (1.02–3.06)	0.043 *
Insecticide use in bedroom (1/0) ^3^	1.75 (1.00–3.06)	0.051	1.83 (1.04–3.20)	0.035 *

^1^ OR refers to odds ratio.^2^ CI refers to confidence interval. ^3^ Reference category: 1 = yes; 0 = no. *p*-value were calculated using a GLMs; * ≤ 0.05.

**Table 8:** GLM of Ln total pyrethroid metabolites (nmole/g creatinine) with one parameter for paired natural log of hand (µg) or surface (µg/m^2^) concentration, household characteristics or insecticide usage parameters (crude models). Adjusted models also include covariates for age, gender, family members involved in agriculture, and distance from home to agricultural area, in addition to the single parameter for hand or surface concentration, household characteristics or insecticide usage (adjusted model). A multivariable model including age, gender, family members involved in agriculture, and distance from home to agricultural area is also presented (*n* = 97).

Parameters	GLM				Multivariable Model		
	Crude OR ^1^ (95% CI ^2^)	*p*-Value	Adjusted OR ^1^ (95% CI ^2^)	*p*-Value	Model	Adjusted OR ^1^ (95% CI ^2^)	*p*-Value
Insecticide storage at home (1/0) ^3^	1.65 (0.96–2.81)	0.068	1.54 (0.90–2.65)	0.118			
Ln permethrin in children’s hand	1.25 (1.10–1.43)	<0.001 ***	1.28 (1.12–1.47)	<0.001 ***	1.	1.38 (1.10–1.44)	0.001 ***
Ln cypermethrin in children’s hand	1.17 (1.00–1.37)	0.049 *	1.18 (1.01–1.38)	0.035 *			
Ln permethrin in floor surface	1.11 (1.02–1.21)	0.013 *	1.12 (1.03–1.22)	0.010 *	2.	1.28 (1.02–1.21)	0.015 *
Ln cypermethrin in floor surface	1.19 (1.06–1.34)	0.004 **	1.20 (1.07–1.36)	0.003 **	3.	1.32 (1.05–1.35)	0.005 **

^1^ OR refers to odds ratio. ^2^ CI refers to confidence interval. ^3^ Reference category: 1 = yes; 0 = no. *p*-value were calculated using a GLM and multivariable model; * ≤ 0.05, ** ≤ 0.01, *** ≤ 0.001.

**Table 9:** GLM of the Ln DCCA (nmole/g creatinine) with one parameter for paired natural log of hand (µg) or surface (µg/m^2^) concentration, household characteristics or insecticide usage parameters (crude models). Adjusted models also include covariates for age, gender, family members involved in agriculture, and distance from home to agricultural area, in addition to the single parameter for hand or surface concentration, household characteristics or insecticide usage (adjusted model). A multivariable model including age, gender, family members involved in agriculture, and distance from home to agricultural area is also presented ( *n* = 97).

Parameters	GLM				Multivariable Model		
	Crude OR ^1^ (95% CI ^2^)	*p*-Value	Adjusted OR ^1^ (95% CI ^2^)	*p*-Value	Model	Adjusted OR ^1^ (95% CI ^2^)	*p*-Value
Insecticide storage at home (1/0) ^3^	1.66 (0.93–2.95)	0.085	1.51 (0.85–2.70)	0.163			
Ln permethrin in children’s hand	1.23 (1.07–1.42)	0.005 **	1.24 (1.07–1.45)	0.005 **	1.	1.32 (1.06–1.43)	0.006 **
Ln cypermethrin in children’s hand	1.19 (1.00–1.40)	0.048 *	1.20 (1.01–1.41)	0.036 *			
Ln permethrin in floor surface	1.10 (1.00–1.20)	0.044 *	1.10 (1.00–1.20)	0.048 *	2.	1.22 (1.00–1.20)	0.049 *
Ln cypermethrin in floor surface	1.18 (1.04–1.34)	0.013 *	1.18 (1.04–1.35)	0.013 *	3.	1.28 (1.03–1.34)	0.016 *

^1^ OR refers to odds ratio. ^2^ CI refers to confidence interval. ^3^ Reference category: 1 = yes; 0 = no. *p*-value were calculated using a GLM and multivariable model; * ≤ 0.05, ** ≤ 0.01.

**Table 10:** GLM of Ln 3-PBA (nmole/g creatinine) with one parameter for paired natural log of hand (µg) or surface (µg/m^2^) concentration, household characteristics or insecticide usage parameters (crude models). Adjusted models also include covariates for age, gender, family members involved in agriculture, and distance from home to agricultural area, in addition to the single parameter for hand or surface concentration, household characteristics or insecticide usage (Adjusted model). A multivariable model including age, gender, family members involved in agriculture, and distance from home to agricultural area is also presented (*n* = 97).

Parameter	GLM				Multivariable Model							
					Model 1		Model 2		Model 3		Model 4	
	Crude OR ^1^ (95% CI ^2^)	*p*-Value	Adjusted OR ^1^ (95% CI ^2^)	*p*-Value	Adjusted OR ^1^ (95% CI ^2^)	*p*-Value	Adjusted OR ^1^ (95% CI ^2^)	*p*-Value	Adjusted OR ^1^ (95% CI ^2^)	*p*-Value	Adjusted OR ^1^ (95% CI ^2^)	*p*-Value
Tile floor (1/0) ^3^	1.69 (1.11–2.57)	0.014 *	1.67 (1.09–2.56)	0.018 *			1.29 (1.13–2.64)	0.012 *	1.22 (1.01–2.34)	0. 034 *	1.26 (1.11–2.50)	0.015 *
Aerosol insecticide sprays (1/0) ^3^	1.59 (1.01–2.50)	0.045 *	1.65 (1.03–2.62)	0.036 *								
Insecticide use in bedroom (1/0) ^3^	1.47 (0.96–2.27)	0.078	1.52 (0.99–2.35)	0.056								
Ln permethrin in children’s hand	1.38 (1.18–1.61)	<0.001 ***	1.45 (1.24–1.71)	<0.001 ***	1.46 (1.18–1.61)	<0.001 ***	NA ^4^	NA ^4^	NA ^4^	NA ^4^	NA ^4^	NA ^4^
Ln cypermethrin in children’s hand	1.21 (1.00–1.46)	0.051	1.22 (1.01–1.47)	0.041 *	NA ^4^	NA ^4^	1.23 (1.01–1.47)	0.038 *	NA ^4^	NA ^4^	NA ^4^	NA ^4^
Ln permethrin in floor surface	1.17 (1.07–1.29)	0.001 **	1.20 (1.08–1.32)	<0.001 ***	NA ^4^	NA ^4^	NA ^4^	NA ^4^	1.33 (1.05–1.28)	0.004 **	NA ^4^	NA ^4^
Ln cypermethrin in floor surface	1.28 (1.11–1.48)	<0.001 ***	1.31 (1.13–1.51)	<0.001 ***	NA ^4^	NA ^4^	NA ^4^	NA ^4^	NA ^4^	NA ^4^	1.38 (1.11–1.47)	<0.001 ***

^1^ OR refers to odds ratio. ^2^ CI refers to confidence interval. ^3^ Reference category: 1 = yes; 0 = no. ^4^ NA refers to not applicable *p*-values were calculated using a GLM and multivariable model; * ≤ 0.05, ** ≤ 0.01, *** ≤ 0.001.

**Table 11:** Health risk assessment from dermal exposure to cypermethrin on the children’s hands was performed using handwipe samples (*n* = 121).

Pesticide	ADD ^1^ (mg/kg/day)	Hazard Quotient (HQ)	Hazard Index (HI)
Permethrin	5.68 × 10^−7^	1.14 × 10^−5^	2.76 × 10^−5^
Cypermethrin	1.63 × 10^−7^	1.63 × 10^−5^	

^1^ ADD refers to average daily dose. Concentration (C) = concentration of contaminant at 95th percentiles in hand wipe sample (mg/cm^2^-event); skin surface area available for contact (SA) = 370 cm^2^ for children aged 3 to <6 years [33]; absorption fraction (ABS) = permethrin 5.7% [34], cypermethrin 10% [35] (unitless); exposure frequency (EF) = 350 days [36]; event frequency (EV) = 1 event/day [36]; exposure duration (ED) = 3.84 years (average age based on questionnaire results); body weight (BW) = 16.57 kg (average weight based on questionnaire results); average time (AT) = ED × 365 days [22].

## Data Availability

The data presented in this study are available on request from the corresponding author.
